# The Hyperæmic Method of Treatment—II

**Published:** 1909-01-23

**Authors:** 


### Orthopædics.

### THE HYPERÆMIC METHOD OF TREATMENT—II.

Hyperæmia by suction is of less general use in orthopædic practice than hyperæmia by constriction. It is, however, applicable to a large number of cases, and as an auxiliary it is exceedingly valuable. Generally, it may be laid down, it is useful in all ulcerated or fistulous lesions. On the neck and on the pelvis the bandage method can scarcely be used. Where lesions exist in such situations the practitioner must resort to the suction cup if he wishes to try the Bier method. Such lesions, however, can rarely be included under the term orthopædics, however widely that word may be stretched. In caries of the cervical spine, and in acute coxalgias, the cups may be useful when applied over the site of tenderness and local pain; but largely the suction method will be limited to fistulous or ulcerating lesions of the extremities. Even in those cases the constriction method will be found extremely useful, but as the practitioner should be thoroughly familiar with the technique of both methods, and as the suction method is of great value in the treatment of other inflammatory surgical lesions, it must be described in full.

Let us suppose that the case for treatment is one of tubercular disease of the metatarsal bones. The affected leg must be imagined to display the usually described features of a fistulous tuberculous osteitis of one or more metatarsals. There is the typical tuberculous sinus on the dorsum of the foot; the patient complains of pain, aggravated at night and by using the foot, and on examination tenderness, fluctuating spots, and necrosed bone, can be felt. Such a case is an excellent one for trying the Bier method by the suction cup.

The foot is carefully washed in a mild antiseptic lotion. All weak granulation tissue is removed, the discharge from the sinus is washed away and the affected spot dried with dossils of gauze or wadding. A cup of the required size is then chosen. Owing to the sharp convexity of the dorsum of the foot this is not easy. The cup must be neither too small nor too large, and its lips must be concave to the requisite degree to enable it to fit easily over the bulge of the dorsum. A proper cup ought to fit easily and smoothly over the lesion, embracing in its mouth not only the sinus and the adjacent area of redness, but half an inch or more of sound and apparently healthy tissue around it. No attempt should be made to apply the cup forcibly. If it has to be pressed deeply down by both hands of the surgeon before it can be made to " grip " at the site where it is proposed to apply it, it should be discarded, for in that case it does not fit properly, and its use is liable to give rise to much pain and great discomfort to the patient. In some—fortunately rare—cases the dorsum of the foot is so much arched that the ordinary foot cups do not fit, and special glasses will have to be ordered. Then it is necessary to measure accurately the degree of arching and in some cases even to take a cast of the foot in order to enable the instrument maker to select a properly fitting cup. In ordinary private practice such cases are, however, very rare, and when they do occur they can always be treated by means of the bandage.

Having chosen the cup, the practitioner attaches it to the rubber tube and exhaust ball, or, if a high degree of suction is required, to an exhaust syringe. Care must be taken before applying the cup to see that the ball or syringe, whichever is selected, works smoothly, and that the tube fits accurately so that no leakage can occur. The rims of the suction glass are then lightly smeared with vaseline or boracic ointment, and the site of application on the dorsum of the foot is ringed with a thin margin of ointment as well. Over this the cup is applied loosely and the ball squeezed tightly until the rims of the cup rest smoothly and accurately on the skin surrounding the sinus. While slight pressure is now made upon the cup the ball is allowed to relax, and the cup will be found to be fixed, more or less securely, over the lesion. It will be noticed that the skin is drawn up into a cone inside the cup, and that this cone rapidly becomes engorged with blood. The condition, in fact, that obtains when the cup is properly applied is exactly the same as in ordinary dry cupping. The foot is allowed to hang down while the patient reclines on a sofa or sits in a chair. The tube and ball are rested on the floor or on a chair, and if the cup has been properly applied there is no fear of it slipping off until it is removed. The writer has found it useful in many cases to let the patients rest the affected foot in a large basin of warm water, on the surface of which the ball and tube float.

How long should the cup be allowed to remain in position? This is a question which can scarcely be answered generally. According to Bier himself, the more acute the lesion, the longer must the cup remain. Thus in a case of a carbuncle (to take a non-orthopædic case) the cup will have to remain in position for 10 or 14 hours, or even longer. In the case of the tubercular foot, cited as an example above, several applications during the day, each lasting from 10 to 15 or 20 minutes, will be found amply sufficient. Too much suction at the first sitting is strongly to be deprecated. It causes pain, and discourages the patient. It is therefore best, even in acute cases, to start with a preliminary application. After a couple of minutes the effect will be easily apparent, and the case must be judged on its individual aspects. Thus, where there is much discharge, the cup will have to be taken off from time to time, cleaned, and replaced. Where much pain results the cause is usually to be found in improperly applied cups; either the cup does not fit or the pressure is too great. In such cases as may be rightly regarded as orthopædic the treatment should be practically painless; what pain attends it is due solely to the dilatation of the vessels and the slight pressure such dilatation may exert on the nerves. In acute conditions, such as fall under the care of the general surgeon, however, the initial pain may be very severe. This is particularly the case in acute septic or phlegmonous inflammations and in large localised suppurations in glandular parts such as the breast, parotid, or testes. When the tendon sheaths are exposed and inflamed the application of the suction method, in the writer's opinion, is not advisable. Usually special apparatus is necessary, and much better results are obtained by the bandage method.

In chronic suppurating cases the patients may be entrusted to apply the cups themselves after they have been taught the simple technique by a few practical demonstrations. They should, however, report themselves frequently, and on such occasions the practitioner would do well himself to apply the cup and see how it fits. As the case progresses to cure, smaller-sized cups become necessary, and when the sinuses are healed these should be wholly discarded in favour of the bandage. After each application the suction cup must be disinfected and thoroughly dried.

With proper care and attention to details the practitioner will find that the method, though it is of limited usefulness when compared with the corresponding bandage method, yields most encouraging results. Tuberculous sinuses of long standing clear up after a few months' steady treatment. As a pain-stilling agent the possibilities of the Bier method are not yet adequately recognised, but anyone endued with a little heroism can convince himself of its excellence in that respect by a simple experiment. This consists in dropping a large lump of melting sealing wax on to the finger nail—a calamity which is not unusual in private dispensing practice—and at once applying the Bier treatment to the injured finger. The pain will be found to vanish almost at once, and the little lesion, which is usually a most inconvenient reminder of an accident for several days afterwards, will be forgotten after a couple of hours. In tuberculous cases the effects of the treatment are almost magical in easing pains. So, too, in acute rheumatic joint pains, in teno-synovitis, and in joint pains generally. In acute suppurations and phlegmonous conditions, however, the treatment at first augments the pain, but after half an hour the patient becomes more comfortable, and gradually the pain subsides altogether.

